# Controle da Pressão Arterial e Fatores Associados em um Serviço Multidisciplinar de Tratamento da Hipertensão

**DOI:** 10.36660/abc.20180384

**Published:** 2020-08-19

**Authors:** Thiago Veiga Jardim, Ana Luiza Lima Souza, Weimar Kunz Sebba Barroso, Paulo Cesar B. Veiga Jardim

**Affiliations:** 1 Universidade Federal de Goiás Programa de Pós-Graduação em Ciências da Saúde Goiânia GO Brasil Universidade Federal de Goiás - Programa de Pós-Graduação em Ciências da Saúde , Goiânia , GO - Brasil; 2 Universidade Federal de Goiás Goiânia GO Brasil Universidade Federal de Goiás - Liga de Hipertensão Arterial, Goiânia , GO - Brasil

**Keywords:** Hipertensão, Pressão Arterial/prevenção e controle, Exercício, Cooperação e Adesão ao Tratamento, Sedentarismo, Obesidade, Estilo de Vida

## Abstract

**Fundamento:**

Apesar de se recomendar a intervenção em equipe no tratamento da hipertensão, resultados dessa abordagem em ambientes do mundo real são escassos na literatura.

**Objetivos:**

Apresentar os resultados de uma estratégia terapêutica baseada em equipe, de longo prazo, de pacientes hipertensos em um serviço de saúde.

**Métodos:**

Dados de pacientes hipertensos acompanhados em um centro de tratamento multidisciplinar localizado na região centro-oeste do Brasil em junho de 2017 com pelo menos duas visitas de acompanhamento foram avaliados retrospectivamente. Dados antropométricos, pressão arterial (PA), tempo de acompanhamento, tratamento farmacológico, diabetes, estilo de vida foram coletados da última consulta. Valores de PA < 140 x 90 mmHg em não diabéticos e < 130 x 80 mmHg em diabéticos foram considerados PA controlada. Um modelo de regressão logística foi construído para identificar variáveis independentemente associadas com o controle da PA. O nível de significância adotado foi de p<0.05.

**Resultados:**

Foram incluídos 1548 pacientes, com média de acompanhamento de 7,6 ± 7,1 anos. A maioria dos pacientes eram mulheres (73,6%; n=1139), com idade média de 61,8 anos. As taxas de controle da PA na amostra total, em não diabéticos e nos diabéticos foram 68%, 79%, e 37,9%, respectivamente. Diabetes associou-se inversamente com controle da PA (OR 0,16; IC95% 0,12-0,20; p<0,001), enquanto idade ≥ 60 anos (OR 1,48; IC95% 1,15-1,91; p=0,003) e sexo feminino (OR 1,38; IC95% 1,05-1,82; p=0,020) apresentaram associação direta.

**Conclusões:**

Uma taxa de controle de cerca de 70% foi encontrada em pacientes atendidos em um serviço multidisciplinar de tratamento da hipertensão. A fim de melhorar esses resultados, atenção deve ser dada a pacientes diabéticos, com idade menor que 60 anos e do sexo masculino. (Arq Bras Cardiol. 2020; 115(2):174-181)

## Introdução

A hipertensão (HTN) pode ser definida como elevação na pressão sanguínea detectada em duas ou mais leituras obtidas em duas ou mais ocasiões, ou uso de medicamentos anti-hipertensivos. ^1 , 2^ Apesar do debate acerca dos limiares a serem adotados para definição da HTN, não há dúvida de que essa condição é um fator de risco cardiovascular e uma causa importante de incapacidade e morte. ^3 - 5^

Pressão arterial (PA) elevada é o fator de risco tratável mais importante de acidente vascular cerebral, fibrilação atrial e insuficiência cardíaca. Reduções na PA são efetivas para prevenir lesões nos órgãos alvo, eventos cardiovasculares e morte em condições clínicas variadas envolvendo diferentes níveis de PA, perfis de risco cardiovascular, e comorbidades. ^6 , 7^ Apesar disso, a HTN não controlada continua uma situação muito prevalente em todo o mundo. ^8^

Entre as estratégias que objetivam melhorar o controle da PA, intervenções em equipe têm se mostrado muito promissoras. ^9 , 10^ Essas estratégias consistem em intervenções organizacionais centradas no paciente, multifacetadas, lideradas por equipes multidisciplinares, que objetivam melhorar a qualidade do cuidado à HTN. O tratamento da HTN em equipe inclui pacientes, profissionais da atenção primária, e outros profissionais, tais como cardiologistas, enfermeiros, farmacêuticos, médicos assistentes, nutricionistas, trabalhadores sociais, profissionais da saúde comunitária, entre outros. Esses profissionais se complementam, oferecendo apoio ao outro e dividindo responsabilidades. ^1^

Apesar do cuidado em equipe ser recomendado para pacientes com HTN pela maioria das diretrizes, ^1 , 2 , 11 , 12^ os resultados dessa intervenção em um ambiente real são escassos na literatura. Conduzimos o presente estudo com o objetivo de relatar os resultados de uma intervenção terapêutica multidisciplinar de longo prazo para pacientes com HTN, com foco na avaliação das taxas de controle da PA e fatores associados.

## Métodos

Dados de todos os pacientes com HTN e idade de 18 anos, com pelo menos duas visitas de seguimento em um centro de tratamento multidisciplinar para HTN na região centro-oeste do Brasil em junho de 2017 foram avaliados retrospectivamente por conveniência.

A HTN foi definida de acordo com a 7ª Diretriz Brasileira de Hipertensão Arterial: (1) PA no consultório ≥ 140 × 90 mmHg; monitorização ambulatorial da PA ≥ 130 × 80 mmHg; (3) monitorização residencial da PA ≥ 135 × 85 mmHg. ^13^ Pacientes em tratamento para HTN também foram considerados hipertensos.

O centro de tratamento em equipe multidisciplinar está em funcionamento por mais de 25 anos, e se dedica ao tratamento de HTN, à educação de profissionais da saúde e à pesquisa. Pacientes com diagnóstico recente de HTN e pacientes com dificuldade de controlar os níveis de PA foram encaminhados para o centro, e o número de pacientes incluídos no estudo foi 1701. A equipe multidisciplinar é composta por médicos (clínicos gerais, cardiologistas, endocrinologistas e nefrologistas), enfermeiros, nutricionistas, terapeutas ocupacionais, educadores físicos, psicólogos e musicoterapeutas. Com o objetivo de melhorar a adesão ao tratamento e reduzir perdas de acompanhamento, o intervalo máximo entre cada visita foi de três meses. O intervalo máximo entre duas consultas médicas foi de seis meses, e em relação a outros profissionais de saúde, não houve visitas de rotina, isto é, as consultas foram agendadas de acordo com as necessidades dos pacientes determinadas por avaliação clínica. Além disso, atividades educacionais e de promoção da saúde foram realizadas a cada duas semanas com os pacientes. ^14 , 15^ Desde o início desse serviço multidisciplinar, as consultas foram registradas em um formulário padronizado. Todos os profissionais diretamente envolvidos no cuidado do paciente foram treinados rotineiramente para o preenchimento desse formulário, assegurando confiabilidade e reprodutibilidade dos dados ao longo dos anos. ^16 , 17^

### Coleta de dados

Foram coletados dados da última visita do paciente, independentemente da especialidade do profissional que o atendeu. Ainda, as datas da primeira consulta foram coletadas e usadas para calcular o período de acompanhamento (diferença entre a primeira e a última visita do serviço), em anos.

Os seguintes dados foram coletados dos prontuários médicos: sexo; idade: em anos, e avaliada pela diferença entre a data de nascimento e a data da última visita; dados antropométricos: peso, altura e Índice de Massa Corporal (IMC), calculado pela fórmula de Quetelet (IMC: peso em Kg/altura ^2^ em metros). O estado nutricional foi classificado de acordo com o IMC, seguindo as definições da Organização Mundial de Saúde: sem sobrepeso (IMC < 25kg/m ^2^ ); com sobrepeso (IMC ≥ 25 kg/m ^2^ e < 30mg/kg ^2^ ) e obeso (IMC ≥ 30 mg/kg ^2^ ).

Pressão arterial: foram realizadas três ou mais medidas da PA, com intervalo mínimo de um minuto. Todas as medidas foram realizadas após cinco minutos de repouso, do membro superior, com o indivíduo sentado e braço apoiado. Foram usados manguitos de tamanho apropriado de acordo com o diâmetro do braço. Os valores médios das duas últimas medidas foram considerados para definição de controle da PA. As medidas foram realizadas com aparelhos oscilométricos (equipamentos semiautomáticos OMRON, modelo HEM-705 CP). Essa rotina foi adotada no serviço para evitar viés de observador.

Estilo de vida: tabagismo (fumante ou não fumante); consumo de bebidas alcóolicas (qualquer consumo relatado na última visita); atividade física de lazer (regular ≥3 vezes por semana), irregular (<3 vezes por semana) e sedentário (nenhuma atividade física).

Diabetes: definida seguindo-se as recomendações das diretrizes mais recentes da Sociedade Brasileira de Diabetes: ^18^ (1) sintomas de poliúria, polidipsia, e perda de peso e glicemia casual (colhida em qualquer horário do dia, independente da última refeição realizada) ≥ 200 mg/dL; (2) glicemia de jejum ≥ 126 mg/dL; o diagnóstico deve ser confirmado repetindo-se a medida em outro dia em caso de pequenas elevações na glicemia; (3) glicemia de duas horas após o teste de tolerância à glicose oral (75g) ≥ 200 mg/dL. O tratamento ao diabetes registrado nos prontuários médicos também foi considerado como critério diagnóstico.

Tratamento medicamentoso: informação sobre tratamento farmacológico para HTN (se o paciente realizava bem como número de medicamentos).

### Definições de controle da PA

Foram adotadas as recomendações da 7ª Diretriz Brasileira de Hipertensão Arterial (2016) ^19^ (valores de PA < 140 x 90 mmHg em não diabéticos e < 130 x 80 mmHg em pacientes diabéticos) para análise do controle da PA.

### Serviço multidisciplinar

Equipe médica: avaliou sintomas, estilo de vida, e medicamentos, realizou exame físico, analisou exames complementares e definiu o tratamento do paciente (prescrição de tratamentos farmacológico e não farmacológico, pedido de exames complementares, e agendamento de visitas de acompanhamento); encaminhou pacientes à emergência ou internação em caso de descompensação clínica aguda.

Enfermeiros: avaliaram sintomas, sinais vitais, estilo de vida e medicamentos; orientaram os pacientes quanto à adesão ao tratamento (farmacológico e não farmacológico); definiram intervalos de retornos com enfermeiro; e encaminharam pacientes para consulta médica se necessário em aspectos clínicos ou para manter um intervalo máximo de seis meses entre duas consultas médicas.

Nutricionistas: enfatizaram aspectos não medicamentosos do cuidado, especificamente a dieta; coletaram dados sobre dieta, avaliaram dados antropométricos e sinais vitais. A abordagem teve como objetivo orientações dietéticas, com ênfase em restrição de sal e prescrição dietética aos pacientes com diagnósticos específicos tais como diabetes e doença renal crônica.

Educadores físicos: promoveram atividade física em grupo para os pacientes (treinamento de resistência a exercício aeróbico) três vezes por semana, e enfatizaram a importância da prática regular de atividade física.

Os demais profissionais da saúde não realizaram visitas formais, e sim uma série de intervenções educacionais para promover a saúde dos pacientes. Os fisioterapeutas conduziram encontros periódicos agendados previamente ou se encontraram com os pacientes na sala de espera e discutiram medidas preventivas contra lesões e quedas. De maneira similar, os psicólogos e os terapeutas musicais atuaram principalmente na sala de espera, fornecendo orientações e intervenções com objetivo de reduzir estresse e melhorar o tempo de espera.

### Análise estatística

A análise estatística foi realizada utilizando-se o programa STATA V14 (StataCorp., College Station, Texas, USA). O teste de Kolmogorov-Smirnov foi usado e confirmou que as variáveis contínuas apresentaram distribuição normal. As variáveis contínuas foram apresentadas em média e desvio padrão, e as variáveis categóricas em número e porcentagem. O teste t não pareado foi usado para comparar as variáveis contínuas e o teste do qui-quadrado para comparar as variáveis categóricas. O modelo de regressão logística foi construído para identificar variáveis com associação independente com o controle da pressão sanguínea. Diabetes, idade ≥ 60 anos, sexo feminino, consumo de bebida alcoólica, tabagismo, sedentarismo, tratamento medicamentoso, IMC, e tempo total de acompanhamento (anos) foram usados como preditores no modelo. O nível de significância adotado foi p<0.05.

## Resultados

Foram incluídos 1548 pacientes no estudo, correspondente a mais de 90% dos pacientes atendidos no serviço (153 não foram incluídos devido a dados faltantes na primeira ou na última consulta). O tempo médio de acompanhamento foi 7,6 ± 7,1 anos. A maioria dos pacientes eram mulheres (73,6%; n=1139), e a média de idade foi 61,8 ±12,8 anos. As mulheres eram mais propensas a serem obesas e sedentárias, mas menos propensas a consumir bebida alcoólica e a fumar em comparação aos homens. Além disso, valores mais baixos de PA foram encontrados em mulheres em comparação a homens. Características da população do estudo, estratificadas por sex, são apresentadas na Tabela 1 .

**Tabela 1:** – Características da população do estudo, estratificadas por sexo (n=1548), Goiânia, Brasil

Fator	Total	Homens	Mulheres	Valor de p*
**N**	1,548 (100%)	409 (26.4%)	1,139 (73.6%)	
**Idade (anos)**	61,8 (±12,8)	62,0 (±13,8)	61,8 (±12,4)	0,750
**Tempo total de acompanhamento (anos)**	7,6 (±7,1)	7,1 (±6,7)	7,8 (±7,2)	0,070
**Altura (m)**	1,58 (±0,09)	1,67 (±0,08)	1,55 (±0,07)	<0,001
**Peso (kg)**	73,8 (±16,5)	79,2 (±16,5)	71,9 (±16,1)	<0,001
**Índice de massa corporal (kg/m** ^**2**^ **)**	29,3 (±5,9)	28,3 (±5,3)	29,7 (±6,0)	<0,001
**Estado nutricional**				
**Sem sobrepeso**	350 (22,6%)	105 (25,7%)	245 (21,5%)	0,084
**Com sobrepeso**	571 (36,9%)	174 (42,5%)	397 (34,9%)	0,006
**Obeso**	627 (40,5%)	130 (31,8%)	497 (43,6%)	<0,001
**Primeira PA sistólica (mmHg)**	146,3 (±24,0)	148,5 (±24,6)	145,5 (±23,8)	0,030
**Primeira PA diastólica (mmHg)**	85,5 (±16,0)	87,2 (±15,6)	84,9 (±16,1)	0,014
**Segunda PA sistólica (mmHg)**	144,5 (±23,1)	146,8 (±23,1)	143,7 (±23,0)	0,018
**Segunda PA diastólica (mmHg)**	83,3 (±13,1)	85,0 (±12,9)	82,7 (±13,1)	0,003
**Terceira PA sistólica (mmHg)**	144,3 (±18,2)	145,1 (±18,4)	144,0 (±18,1)	0,320
**Terceira PA diastólica (mmHg)**	83,2 (±10,2)	84,4 (±10,4)	82,8 (±10,1)	0,009
**PA sistólica média (mmHg)** ^**†**^	144,4 (±19,1)	145,9 (±19,4)	143,8 (±18,9)	0,057
**PA diastólica média (mmHg)** ^**†**^	83,3 (±10,6)	84,7 (±10,7)	82,8 (±10,6)	0,002
**Diabetes**	412 (26,6%)	113 (27,6%)	299 (26,3%)	0,590
**Consumo de álcool**	206 (13,3%)	108 (26,4%)	98 (8,6%)	<0,001
**Tabagismo**	177 (11,4%)	73 (17,8%)	104 (9,1%)	<0,001
**Atividade física**				
**Sedentarismo**	737 (47,6%)	172 (42,1%)	565 (49,6%)	0,009
**Irregular**	231 (14,9%)	70 (17,1%)	161 (14,1%)	0,150
**Regular**	580 (37,5%)	167 (40,8%)	413 (36,3%)	0,100
**Tratamento farmacológico**	1,513 (97,7%)	399 (97,6%)	1,114 (97,8%)	0,770
**Número de drogas anti-hipertensivas**	2,1 (± 0,8)	2,8 (± 0,7)	1,7 (± 0,8)	0,369

Valores em média (±DP) ou n (%); PA: pressão arterial; * teste t não pareado para comparar variáveis contínuas e teste do qui-quadrado para comparar variáveis categóricas; significância estatística α<0,05. †média da segunda e da terceira leitura

**Figura 1:**
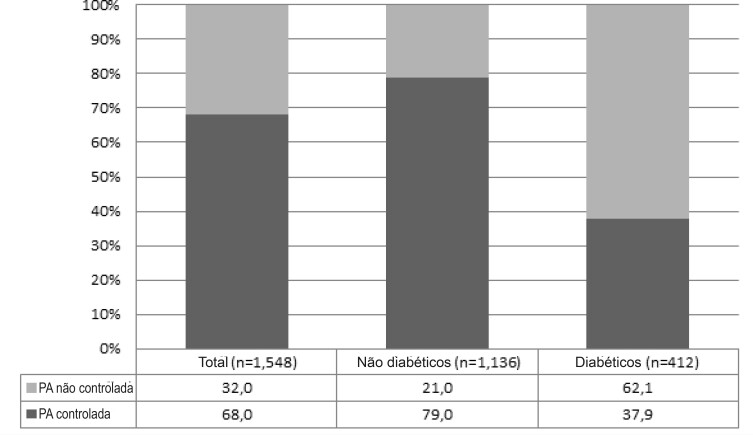
– Controle da pressão arterial na população total estudada, e em diabéticos e não diabéticos; Goiânia, Brasil. PA: pressão arterial; controle da pressão arterial: PA < 140 x 90 mmHg em não diabéticos e < 130 x 80 mmHg em diabéticos.

A taxa de controle da PA na população do estudo foi de 68%, e esse valor foi mais alto quando somente pacientes não diabéticos foram considerados (79%). Por outro lado, avaliando-se exclusivamente pacientes diabéticos, a taxa de controle da PA caiu para 37.9%. A Figura 1 apresenta um resumo das taxas de controle da PA em nosso estudo.

Indivíduos com PA sob controle tendiam a ser do sexo feminino, mais velhas, com períodos mais longos de acompanhamento e IMC mais baixo em comparação àqueles com PA não controlada. Ainda, aqueles com PA controlada eram menos propensos a serem obesos, diabéticos e sedentários comparativamente àqueles com PA controlada. As características da população do estudo, estratificadas por controle de PA, são apresentadas na Tabela 2 .

**Tabela 2:** – Características da população do estudo segundo controle ou não da pressão arterial * (n=1548), Goiânia, Brasil

Fator	PA controlada	PA não controlada	Valor de p†
**N**	1,053	495	
**Sexo feminino**	793 (75,3%)	346 (69,9%)	0,024
**Idade (anos)**	62,8 (±13,1)	59,8 (±11,9)	<0,001
**Tempo de acompanhamento (anos)**	8,1 (±7,4)	6,6 (±6,5)	<0,001
**Altura (m)**	1,58 (±0,09)	1,59 (±0,09)	0,059
**Peso (kg)**	72,4 (±16,6)	76,9 (±15,9)	<0,001
**Índice de massa corporal (kg/m** ^**2**^ **)**	28,9 (±5,8)	30,4 (±6,0)	<0,001
**Estado nutricional**			
**Sem sobrepeso**	262 (24,9%)	88 (17,8%)	0,002
**Com sobrepeso**	399 (37,9%)	172 (34,7%)	0,230
**Obeso**	392 (37,2%)	235 (47,5%)	<0,001
**Primeira PA sistólica (mmHg)**	138,8 (19,8)	162,2 (24,4)	<0,001
**Primeira PA diastólica (mmHg)**	80,6 (14,7)	95,9 (13,4)	<0,001
**Segunda PA sistólica (mmHg)**	137,0 (18,8)	160,3 (23,4)	<0,001
**Segunda PA diastólica (mmHg)**	77,7 (9,9)	95,2 (11,0)	<0,001
**Terceira PA sistólica (mmHg)**	139,4 (15,8)	154,7 (18,5)	<0,001
**Terceira PA diastólica (mmHg)**	79,58 (7,8)	91,3 (10,1)	<0,001
**PA sistólica média (mmHg)** ^**† †**^	138,2 (±15,8)	157,5 (±18,9)	<0,001
**PA diastólica média (mmHg)** ^**† †**^	78,6 (±7,7)	93,2 (±9,0)	<0,001
**Diabetes**	156 (14,8%)	256 (51,7%)	<0,001
**Consumo de álcool**	130 (12,3%)	76 (15,4%)	0,100
**Tabagismo**	119 (11,3%)	58 (11,7%)	0,810
**Atividade física**			
**Sedentarismo**	479 (45,5%)	258 (52,1%)	0,015
**Irregular**	163 (15,5%)	68 (13,7%)	0,370
**Regular**	411 (39,0%)	169 (34,1%)	0,064
**Tratamento farmacológico**	1,028 (97,6%)	485 (98,0%)	0,660
**Número de drogas anti-hipertensivas**	3,00 (± 0,81)	2,81 (± 0,76)	0,432

Valores dados em médias (±DP) ou n (%). *Controle da pressão arterial (PA) - PA <140 x 90 mmHg em não diabéticos e < 130 x 80 mmHg em diabéticos. † teste t não pareado para comparações das variáveis contínuas e teste do qui-quadrado para comparações das variáveis categóricas; significância estatística α<0,05. ‡média da segunda e da terceira leitura

O modelo de regressão logística multivariada usado para identificar variáveis independentemente associadas com o controle da PA nessa população mostrou que diabetes foi inversamente associado, enquanto idade ≥ 60 anos e sexo feminino apresentaram associação direta com controle da PA ( Tabela 3 ).

**Tabela 3:** – Variáveis independentemente associadas com controle da pressão arterial (n=1548); Goiânia – Brasil

Variáveis	Odds Ratio	[Intervalo de confiança 95%]	Valor de p
**Diabetes**	0,15	[0,11-0,20]	<0,001
**Idade ≥ 60 anos**	1,45	[1,13-1,90]	0,005
**Sexo feminino**	1,36	[1,09-1,88]	0,022
**Consumo de álcool**	0,80	[0,56-1,15]	0,183
**Tabagismo**	1,25	[0,80-1,80]	0,330
**Sedentarismo**	0,78	[0,60-1,02]	0,053
**Tratamento farmacológico**	1,12	[0,50-2,47]	0,741
**Índice de massa corporal (Kg/m** ^**2**^ **)**	0,97	[0,95-1,01]	0,088
**Tempo de acompanhamento (anos)**	1,01	[1,00-1,03]	0,098
**Número de drogas anti-hipertensivas**	0,85	[0,68-1,01]	0,320

## Discussão

Nós avaliamos os dados de mais de 1500 pacientes hipertensos acompanhamentos regularmente em um serviço de cuidado multidisciplinar para mostrar os resultados dessa abordagem em equipe em uma situação real. Todos os pacientes incluídos no estudo foram encaminhados para um centro especializado no tratamento da hipertensão e tiveram o tratamento coberto pelo Sistema Único de Saúde. Ainda, as características basais dos pacientes eram similares àquelas descritas no Registro Brasileiro de Hipertensão Arterial, ^20^ mostrando a capacidade de se generalizar os resultados deste estudo. Quase 70% dos pacientes apresentavam a PA controlada, e essa taxa subiu para 79% quando somente pacientes não diabéticos foram considerados. O controle da PA foi inversamente associado com diabetes e diretamente associado com idade ≥ 60 anos e sexo feminino.

Estudos populacionais conduzidos no Brasil mostraram que as taxas de controle da PA variaram de 10,1% a 57,6%, dependendo da região do país e características dos pacientes. ^21^ Nenhum desses estudos, no entanto, utilizou dados obtidos de centros de tratamento em equipe. Nossa taxa de controle da PA global (68%) foi maior que as obtidas por tratamento convencionais. Em comparação às taxas de controle da PA relatadas em outros países de renda média como a África do Sul (30 e 49%) ^22 , 23^ e mesmo a um país de alta renda como os Estados Unidos (48%), ^24^ nós encontramos resultados melhores com intervenção baseada em equipe no estudo atual.

O controle da PA em pacientes com HTN e diabetes é um desafio; as taxas de controle são geralmente mais baixas em comparação a de pacientes hipertensos sem diabetes. ^25^ Além disso, pacientes diabéticos hipertensos têm maior chance de desenvolverem HTN resistente verdadeira. ^26^ Somente 37,9% de nossos pacientes diabéticos hipertensos apresentavam PA controlada, em oposição a 79% entre pacientes não diabéticos. Ainda, o diabetes apresentou associação inversa e independente com o controle da PA nesta abordagem terapêutica multidisciplinar.

Idade avançada tem sido associada com controle da PA em diferentes populações. ^22 , 27^ Nossos resultados reforçam esse dado, uma vez que idade ≥ 60 anos apresentou associação direta com o controle da PA. Além disso, nosso estudo é original ao apresentar uma associação entre idades mais avançadas e controle da PA em um abordagem de tratamento baseado em equipe.

Diferença na taxa de controle da PA entre sexo é controversa. Enquanto alguns estudos apontam que mulheres têm maior probabilidade de apresentarem HTN não controlada em comparação a homens, ^28^ outros indicaram uma associação entre sexo feminino e controle adequado da PA. ^22^ Em nosso serviço de equipe multidisciplinar, esta é a primeira vez que os resultados apontam para uma associação direta entre sexo feminino e melhores taxas de controle da PA. ^16 , 17^

Ensaios clínicos randomizados são geralmente considerados a melhor evidência cientifica para se confirmar ou não a eficácia e a segurança de um tratamento. ^29 , 30^ Uma vez que há evidências disponíveis e diretrizes recomendam tratamentos, é importante avaliar o desempenho dessas intervenções na vida real. A realidade do cuidado ao paciente em um ensaio clínico randomizado é diferente da prática clínica diária em muitos aspectos ^31^ Nesse sentido, os resultados positivos apresentados aqui, particularmente considerando que nosso estudo foi realizado em um serviço de saúde público de um país com recursos limitados, reforçam a importância de uma abordagem baseada em equipe no tratamento da hipertensão.

O delineamento do estudo pode ser considerado como uma limitação, uma vez que realizamos um estudo retrospectivo unicêntrico, sem grupo controle. Apesar disso, todos os prontuários médicos são objetivos e seu preenchimento exaustivamente treinado nesse centro, contribuindo para a confiabilidade dos dados. Ainda, apesar de sabermos que o uso de um grupo controle seria mais apropriado, os resultados positivos aqui encontrados podem ser usados como base para estudos futuros e ajudar como informação aos profissionais de saúde sobre uma estratégia bem sucedida no manejo de pacientes com HTN.

Outra limitação potencial refere-se à avaliação da atividade física. Somente atividade física formal ou planejada – caminhada, corrida, ciclismo, natação, treinamento de força, etc. – foi considerada na análise em nosso estudo. Assim, as atividades físicas diárias não foram consideradas e, portanto, os resultados de estilo de vida sedentário podem ter sido superestimados.

Custos de implementação e manutenção devem ser considerados no tratamento em equipe da hipertensão. Apesar disso, a avaliação econômica dessa intervenção em países de alta renda mostrou que a abordagem multidisciplinar para melhorar o controle da PA é custo efetiva. ^32^ As mesmas avaliações devem ser realizadas em países de renda média-baixa.

Dados os resultados positivos do presente estudo e estudos prévios envolvendo pacientes do mesmo centro de tratamento da HTN, ^14 , 16 , 17 , 33 , 34^ o formato adotado em nosso serviço pode ser um modelo para outros centros de tratamento de pacientes com HTN que almejam implementar uma estratégia de tratamento em equipe.

## Conclusão

No presente estudo, conduzido em um ambiente real, a taxa de controle da PA após uma abordagem baseada em equipe a pacientes hipertensos foi 70%. A fim de melhorar esses resultados, atenção deve ser dada a pacientes diabéticos, com idade menor que 60 anos e do sexo masculino.
