# Supplementary material for: Controle da Pressão Arterial e Fatores Associados em um Serviço Multidisciplinar de Tratamento da Hipertensão
Source: Arq Bras Cardiol. 2020 Aug 19;115(2):174–81. [Article in Portuguese] doi: 10.36660/abc.20180384 (PMC8384292; doi:10.36660/abc.20180384)
Supplement: Translation

# Translation: Blood Pressure Control and Associated Factors in a Real-World Team-Based Care Center

**Authors:** Thiago Veiga Jardim, Ana Luiza Lima Souza, Weimar Kunz Sebba Barroso, Paulo Cesar B. Veiga Jardim

**Affiliations:** 1 Brazil Universidade Federal de Goiás - Programa de Pós-Graduação em Ciências da Saúde, Goiânia, GO - Brazil; 2 Brazil Universidade Federal de Goiás - Liga de Hipertensão Arterial, Goiânia, GO - Brazil

**Keywords:** Hypertension, Blood Pressure/prevention and control, Exercise, Treatment Adherence and Compliance, Sedentarism, Obesity, Life Style

## Abstract

**Background:**

Although team-based care is recommended for patients with hypertension, results of this intervention in a real-world setting are missing in the literature.

**Objective:**

To report the results of a real-world long-term team-based care for hypertensive patients we conducted this study.

**Methods:**

Data of hypertensive patients attending a multidisciplinary treatment center located in the Midwest region of Brazil in June 2017 with at least two follow-up visits were retrospectively assessed. Anthropometric, blood pressure (BP), follow-up time, pharmacological treatment, diabetes and lifestyle data were collected from the last visit to the service. BP values < 140 x 90 mmHg in non-diabetics and < 130 x 80 mmHg in diabetics were considered controlled. A logistic regression model was built to identify variables independently associated to BP control. Significance level adopted p < 0.05.

**Results:**

A total of 1,548 patients were included, with a mean follow-up time of 7.6 ± 7.1 years. Most patients were female (73.6%; n=1,139) with a mean age of 61.8 ±12.8 years. BP control rates in all the sample, and in non-diabetics and diabetics were 68%, 79%, and 37.9%, respectively. Diabetes was inversely associated with BP control (OR 0.16; 95%CI 0.12-0.20; p<0.001) while age ≥ 60 years (OR 1.48; 95%CI 1.15-1.91; p=0.003) and female sex (OR 1.38; 95%CI 1.05-1.82; p=0.020) were directly associated.

**Conclusions:**

A BP control rate around 70% was found in patients attending a multidisciplinary team care center for hypertension. Focus on patients with diabetes, younger than 60 years and males should be given to further improve these results. (Arq Bras Cardiol. 2020; 115(2):174-181)

## Introduction

Hypertension (HTN) is defined as elevated blood pressure (BP) levels based on an average of ≥ two careful readings obtained on ≥ two occasions, or current use of BP-lowering medications.^1 , 2^ Although there is some debate on which thresholds should be used to define HTN, there is no doubt about the burden of HTN as a cardiovascular risk factor and a major cause of disability and death.^3 - 5^

Elevated BP is the most important treatable risk factor for stroke, atrial fibrillation and heart failure.^5^ Reductions in BP are effective to prevent target organ damage, cardiovascular events and death in various clinical conditions involving different BP levels, cardiovascular risk profiles, and comorbidities.^6 , 7^ Despite that, uncontrolled HTN remains a widely prevalent situation worldwide.^8^

Among the strategies aimed to improve BP control, team-based interventions have shown to be highly promising.^9 , 10^ They consist of organizational, patient-centered, multifaceted interventions, led by multidisciplinary teams, aimed at improving the quality of HTB care. HTN team-based care includes patients, patient’s primary care providers, and other professionals, such as cardiologists, nurses, pharmacists, physician assistants, dietitians, social workers, community health workers, and others. These workers complement each other by providing process support and sharing responsibilities.^1^

Although team-based care is recommended for patients with HTN by most guidelines,^1 , 2 , 11 , 12^ results of this intervention in a real-world setting are missing in the literature. We conducted this study aiming to report the results of a long-term multidisciplinary treatment intervention for patients with HTN, specifically assessing BP control rates and associated factors.

## Methods

Data of all patients with HTN aged 18 years or older, with at least two follow-up visits and attending a multidisciplinary treatment center for HTN in the Central West region of Brazil in June 2017 were retrospectively assessed by convenience.

HTN was defined according to the 7th Brazilian Guidelines on Hypertension: (1) office BP ≥ 140 × 90 mmHg; ambulatory BP monitoring ≥ 130 × 80 mmHg; (3) home BP monitoring ≥ 135 × 85 mmHg.^13^ Patients receiving HTN treatment were also considered hypertensive.

The center has been functioning for more than 25 years, dedicated to the treatment of HTN, health professional education and research. Patients with recently diagnosed HTN or those with difficulties to control BP levels are referred to the center and the total number of patients enrolled in the study was 1,701. The multidisciplinary team consists of physicians (general practitioners, cardiologists, endocrinologists and nephrologists), nurses, dietitians, physical therapists, physical educators, psychologists and musical therapists. Aiming to improve treatment compliance and reduce loss of follow-up, the maximum interval between each patient appointment was three months. The maximum interval between two medical visits was six months, and regarding the other healthcare professionals, there was no routine appointments, *i.e* ., the visits were scheduled according to patient’s needs determined by clinical examination. Additionally, educational and health promotion activities were performed every two weeks with patients.^14 , 15^ Since beginning of this multidisciplinary service, consultations have been registered in a standardized form. All healthcare professionals directly involved in patients’ care were routinely trained to complete this form, ensuring data reliability and reproducibility throughout years.^16 , 17^

### Data collection

Data of the last visit to the service were collected, regardless of the healthcare specialty. Additionally, the dates of patient’s first visit registered in medical charts were collected and used to calculate the follow-up time (difference between the first and the last visit to the service), in years.

The following data were collected from the medical records: sex; age: given in years and assessed by the difference between birth date and date of last visit; anthropometric data: weight, height and body mass index (BMI) calculated using the Quetelet formula (BMI = weight in kg/height^2^in meter). Nutritional status was classified according to BMI and following the World Health Organization definitions: non-overweight (BMI < 25kg/m^2^); overweight (BMI ≥ 25 kg/m^2^and < 30mg/kg^2^) and obese (BMI ≥ 30 mg/kg^2^).

BP: a minimum of three BP measurements, with at least 1-min interval, was taken. All measurements were performed after 5 minutes of rest, on the upper limb, with the individual sitting and the arm supported. Appropriate cuffs were used depending on arm diameter. Mean values of the last two measurements were considered for BP control definition. BP measurements were performed with oscillometric devices (OMRON semi-automatic equipment, model HEM-705CP). This routine was adopted in the service to avoid observer bias.

Lifestyle: smoking (current smoker or nonsmoker); alcohol consumption (any alcohol consumption reported during the last visit); leisure physical activity (regular: ≥3 times a week, irregular: <3 times a week and sedentary: no activity).

Diabetes: definition followed the recommendations of the most recent guidelines of the Brazilian Society of Diabetes:^18^ (1) symptoms of polyuria, polydipsia, weight loss and casual blood glucose (values obtained at any time of the day regardless of meal times) ≥ 200 mg/dL; (2) fasting blood glucose ≥ 126 mg/dL; diagnosis should be confirmed by repeat testing on another day in case of small blood sugar elevations; (3) 2-hour plasma glucose value after a 75-g oral glucose tolerance test ≥ 200 mg/dL. Diabetes treatment registered in medical records was also considered as diagnosis criteria.

Drug treatment: information whether patient was receiving or not pharmacological HTN treatment and the number of antihypertensive medications.

### BP control definitions

Recommendations of the 7th Brazilian Guidelines on hypertension (2016)^19^ were adopted (BP values < 140 x 90 mmHg in non-diabetics and < 130 x 80 mmHg in diabetic patients) for analysis of BP control.

### Multidisciplinary service

Medical team: assessed symptoms, lifestyle habits and use of medications; performed patients’ physical examination; analyzed complementary tests and established patient management (pharmacological and nonpharmacological treatments prescription, complementary tests request, and follow-up visits schedule); referred patients to emergency care or hospitalization if acute clinical decompensation was identified.

Nurses: assessed symptoms, vital signs, lifestyle habits and use of medications; instructed about compliance to both pharmacological and nonpharmacological treatments; defined intervals of visits to the nurse; and referred patients for medical consultation if clinically necessary or to ensure a maximum interval of six months between two medical visits.

Dietitians: emphasized nonpharmacological aspects of care, specifically the diet; collected dietary data; assessed anthropometric data and vital signs. Management was aimed at dietary guidance with emphasis on salt restriction and prescription of diets for patients with specific diagnosis such as diabetes and chronic kidney disease.

Physical educators: developed and assist patients in group physical activities (strength training and aerobic exercise) three times a week and emphasized the importance of regular physical activity.

The other health care professionals did not conduct formal appointments, but rather a series of educational interventions to promote patients’ health. Physical therapists conducted periodical meetings previously scheduled or saw with patients at the waiting room and discussed preventive measures for injuries and falls. Similarly, psychologists and musical therapists acted mainly in the waiting room, providing instructions and interventions aimed at stress reduction and improve the waiting time.

### Statistical analysis

Statistical analysis was performed using the software STATA V14 (StataCorp., College Station, Texas, USA). The Kolmogorov-Smirnov test was used and determined that the continuous variables were normally distributed. Continuous variables are presented as mean and standard deviation. Categorical variables are presented as n and %. Unpaired T-test was used to compare continuous variables and the chi-square test to compare categorical ones. A logistic regression model was built to identify variables independently associated to blood pressure control. Diabetes, age ≥ 60 years, female sex, alcohol consumption, smoking, sedentary lifestyle, pharmacological treatment, BMI (kg/m^2^) and total follow-up time (years) were used as predictors in the model. The significance level adopted was p < 0.05.

## Results

A total of 1,548 patients were included, accounting for more than 90% of all patients attending the center (153 were not included due to missing data on the first or last visits). Mean follow-up time was 7.6 (±7.1) years. Most patients were female (73.6%; n=1,139) and the mean age was 61.8 (±12.8) years. Women were more likely to be obese and sedentary, while less likely to consume alcohol and smoke when compared to men. Additionally, lower BP values were found in females when compared to males. Characteristics of the study population, stratified by sex, are presented in Table 1 .

**Table 1:** – Characteristics of the study population stratified by sex (n=1,548), Goiânia, Brazil

Factor	Overall	Male	Female	p-value^*^
**N**	1,548 (100%)	409 (26.4%)	1,139 (73.6%)	
**Age (years)**	61.8 (±12.8)	62.0 (±13.8)	61.8 (±12.4)	0.750
**Total follow-up time (years)**	7.6 (±7.1)	7.1 (±6.7)	7.8 (±7.2)	0.070
**Height (m)**	1.58 (±0.09)	1.67 (±0.08)	1.55 (±0.07)	<0.001
**Weight (kg)**	73.8 (±16.5)	79.2 (±16.5)	71.9 (±16.1)	<0.001
**Body mass index (kg/m** ^**2**^ **)**	29.3 (±5.9)	28.3 (±5.3)	29.7 (±6.0)	<0.001
**Nutritional status**				
**Non-overweight**	350 (22.6%)	105 (25.7%)	245 (21.5%)	0.084
**Overweight**	571 (36.9%)	174 (42.5%)	397 (34.9%)	0.006
**Obese**	627 (40.5%)	130 (31.8%)	497 (43.6%)	<0.001
**First systolic BP (mmHg)**	146.3 (±24.0)	148.5 (±24.6)	145.5 (±23.8)	0.030
**First diastolic BP (mmHg)**	85.5 (±16.0)	87.2 (±15.6)	84.9 (±16.1)	0.014
**Second systolic BP (mmHg)**	144.5 (±23.1)	146.8 (±23.1)	143.7 (±23.0)	0.018
**Second diastolic BP (mmHg)**	83.3 (±13.1)	85.0 (±12.9)	82.7 (±13.1)	0.003
**Third systolic BP (mmHg)**	144.3 (±18.2)	145.1 (±18.4)	144.0 (±18.1)	0.320
**Third diastolic BP (mmHg)**	83.2 (±10.2)	84.4 (±10.4)	82.8 (±10.1)	0.009
**Mean systolic BP (mmHg)** ^**†**^	144.4 (±19.1)	145.9 (±19.4)	143.8 (±18.9)	0.057
**Mean diastolic BP (mmHg)** ^**†**^	83.3 (±10.6)	84.7 (±10.7)	82.8 (±10.6)	0.002
**Diabetes**	412 (26.6%)	113 (27.6%)	299 (26.3%)	0.590
**Alcohol consumption**	206 (13.3%)	108 (26.4%)	98 (8.6%)	<0.001
**Smoking**	177 (11.4%)	73 (17.8%)	104 (9.1%)	<0.001
**Physical activity**				
**Sedentary**	737 (47.6%)	172 (42.1%)	565 (49.6%)	0.009
**Irregular**	231 (14.9%)	70 (17.1%)	161 (14.1%)	0.150
**Regular**	580 (37.5%)	167 (40.8%)	413 (36.3%)	0.100
**Pharmacological treatment**	1,513 (97.7%)	399 (97.6%)	1,114 (97.8%)	0.770
**Number of anti- hypertensive drugs**	2.1 (± 0.8)	2.8 (± 0.7)	1.7 (± 0.8)	0.369

Values given in means (±SD) or n (%).^*^ unpaired t-test to compare continuous variables and chi-square test for comparison of categorical variables; statistically significant at α<0.05.^†^mean value of second and third readings .

BP control rate in the study population was 68%, and this value was higher when only non-diabetic patients were considered (79%). On the other hand, assessing exclusively diabetic patients, BP control rate dropped to 37.9%. Figure 1 shows a summary of BP control rates in our study.

**Figure 1:** – Blood pressure control in the overall study population, non-diabetics and diabetics. Goiânia – Brazil. Blood pressure control – BP < 140 x 90 mmHg in non-diabetics and < 130 x 80 mmHg in diabetics.

Individuals with BP under control were more likely to be females, older, with longer follow-up periods and lower BMI when compared to those with uncontrolled BP. Additionally those with controlled BP were less likely to be obese, diabetic and sedentary in comparison to those without BP controlled. Characteristics of the study population, stratified by BP control, are presented in Table 2 .

**Table 2:** – Study population characteristics by blood pressure control* (n=1,548). Goiânia – Brazil

Factor	Controlled	Uncontrolled	p-value†
**N**	1,053	495	
**Female sex**	793 (75.3%)	346 (69.9%)	0.024
**Age (years)**	62.8 (±13.1)	59.8 (±11.9)	<0.001
**Total follow-up time (years)**	8.1 (±7.4)	6.6 (±6.5)	<0.001
**Height (m)**	1.58 (±0.09)	1.59 (±0.09)	0.059
**Weight (kg)**	72.4 (±16.6)	76.9 (±15.9)	<0.001
**Body mass index (kg/m2)**	28.9 (±5.8)	30.4 (±6.0)	<0.001
**Nutritional status**			
**Non-overweight**	262 (24.9%)	88 (17.8%)	0.002
**Overweight**	399 (37.9%)	172 (34.7%)	0.230
**Obese**	392 (37.2%)	235 (47.5%)	<0.001
**First systolic BP (mmHg)**	138.8 (19.8)	162.2 (24.4)	<0.001
**First diastolic BP (mmHg)**	80.6 (14.7)	95.9 (13.4)	<0.001
**Second systolic BP (mmHg)**	137.0 (18.8)	160.3 (23.4)	<0.001
**Second diastolic BP (mmHg)**	77.7 (9.9)	95.2 (11.0)	<0.001
**Third systolic BP (mmHg)**	139.4 (15.8)	154.7 (18.5)	<0.001
**Third diastolic BP (mmHg)**	79.58 (7.8)	91.3 (10.1)	<0.001
**Mean systolic BP (mmHg)** ^**‡**^	138.2 (±15.8)	157.5 (±18.9)	<0.001
**Mean diastolic BP (mmHg)** ^**‡**^	78.6 (±7.7)	93.2 (±9.0)	<0.001
**Diabetes**	156 (14.8%)	256 (51.7%)	<0.001
**Alcohol consumption**	130 (12.3%)	76 (15.4%)	0.100
**Smoking**	119 (11.3%)	58 (11.7%)	0.810
**Physical activity**			
**Sedentary**	479 (45.5%)	258 (52.1%)	0.015
**Irregular**	163 (15.5%)	68 (13.7%)	0.370
**Regular**	411 (39.0%)	169 (34.1%)	0.064
**Pharmacological treatment**	1,028 (97.6%)	485 (98.0%)	0.660
**Number of anti-hypertensive drugs**	3.00 (± 0.81)	2.81 (± 0.76)	0.432

Values given in means (±SD) or n (%). *Blood pressure control - BP <140 x 90 mmHg in non-diabetics and < 130 x 80 mmHg in diabetics. † unpaired T-test to compare continuous variables and Chi Square test to compare categorical ones; statistically significant at α<0.05. ‡mean value of second and third readings.

The multivariable logistic regression model built to identify variables independently associated to BP control in this population showed that diabetes was inversely associated with BP control while age ≥ 60 years and female sex were directly associated ( Table 3 ).

**Table 3:** – Variables independently associated to blood pressure control (n=1,548). Goiânia – Brazil

Variables	Odds Ratio	[95% Conf.Interval]	p-value
Diabetes	0.15	[0.11-0.20]	<0.001
Age ≥ 60 years	1.45	**[** 1.13-1.90 **]**	0.005
Female sex	1.36	**[** 1.09-1.88 **]**	0.022
Alcohol consumption	0.80	**[** 0.56-1.15 **]**	0.183
Smoking	1.25	**[** 0.80-1.80 **]**	0.330
Sedentary lifestyle	0.78	**[** 0.60-1.02 **]**	0.053
Pharmacological treatment	1.12	**[** 0.50-2.47 **]**	0.741
Body mass index (kg/m2)	0.97	**[** 0.95-1.01 **]**	0.088
Total follow-up time (years)	1.01	**[** 1.00-1.03 **]**	0.098
Number of anti-hypertensive drugs	0.85	[0.68-1.01]	0.320

## Discussion

We assessed data of more than 1,500 hypertensive patients with regular follow-up in a team-based care center to show the results of this multidisciplinary therapeutic strategy in a real-world setting. All patients included in this study were referred to a center specialized in hypertension treatment and had their treatment fully covered by Brazil’s universal health system. Additionally, baseline characteristics of the patients were similar to those reported in the Brazilian Registry of Hypertension,^20^ showing the generalizability of the results of the study. Almost 70% of the all patients had their BP controlled, and those results went up to 79% considering only the non-diabetic patients. BP control was inversely associated with diabetes and directly associated with age ≥ 60 years and female sex.

Population studies conducted in Brazil showed that BP control rates varied from 10.1% to 57.6% depending on country region and patient characteristics.^21^ None of these studies, however, used data from team-based care centers. Our overall control rate (68%) was higher than those reported in conventional treatments in Brazil. As compared to BP control rates reported in other middle income countries like South Africa (30 and 49%),^22 , 23^ and even in a high-income country like the United States of America (48%),^24^ in the current study, we found better results with a team-based intervention.

BP control in patients with HTN and diabetes is challenging; control rates are usually lower than the ones found in hypertensive patients without diabetes.^25^ Also, diabetic hypertensive patients are more likely to develop resistant hypertension.^26^ Only 37.9% of our diabetic hypertensive patients had their BP under control as opposed to the 79% control rate among the non-diabetic patient. Additionally, diabetes was independently and inversely associated to BP control in this team-based care setting.

Older ages have been associated to BP control in different populations.^22 , 27^ Our results reinforce these findings since we found that age ≥ 60 years was directly associated to BP control. Besides that, the novelty of our findings is the association between older ages and BP control in a team-based care strategy.

Sex differences in BP control rates are controversial. While studies have reported that women are more likely than men to have uncontrolled HTN,^28^ others have indicated an association between female sex and appropriate hypertension management.^22^ In our team-based care center, this is the first time that female sex is directly associated with higher HTN control rates.^16 , 17^

Randomized controlled trials are often considered the best scientific evidence for ascertaining efficacy and safety of a treatment.^29 , 30^ Once the evidence is available and guidelines recommend treatments, it is important to assess how such interventions perform in a real-world setting. After all, the reality of patient care in a randomized clinical trial is different from usual clinical practice in many ways.^31^ In that sense, the positive results shown here, particularly considering that our study was conducted in a public healthcare setting from a country with limited resources, reinforce the relevance of team-based care on hypertension management.

The study design might be a limitation, since we conducted a retrospectively single center study with no control group. Despite that, all medical records are objective, and their completion is exhaustively trained in this center, contributing to reliability of the data. Additionally, although we acknowledge that using a control group would be more appropriate, the positive result found here can foster future studies and help informing the healthcare community about a successful way to manage patients with HTN.

Another potential limitation regards to physical activity assessment. Only planned or formal physical activity – walking, running, cycling, swimming, strength training, etc.), was included in our definition. Therefore, daily physical activities were not considered and our sedentary lifestyle results are probably overestimated.

Costs of implementation and maintenance need to be taken into account when considering a team-based care for hypertension management. Despite that, economic assessment of this intervention in high-income countries showed that team-based care to improve BP is cost-effective.^32^ Same assessments need to be conducted in low-to-middle income countries.

Given the positive results of the present study and previous studies involving patients from the same HTN treatment center,^14 , 16 , 17 , 33 , 34^ the format adopted in our service can be a model for other centers handling patients diagnosed with HTN and aiming to implement a team-based strategy.

## Conclusion

In the present study, conducted in a real-world setting, the rate of BP control after a team-based approach to hypertensive patients was 70%. Focus on patients with diabetes, younger than 60 years and males should be given to further improve these results.
